# Adolescents with autism spectrum disorder exhibit intact physical causal inference but weak intention inference

**DOI:** 10.3389/fpsyg.2024.1458101

**Published:** 2024-10-24

**Authors:** Meng-Jung Liu

**Affiliations:** Department of Special Education, National Kaohsiung Normal University, Kaohsiung, Taiwan

**Keywords:** autism spectrum disorder, theory of mind, mentalizing, intention attribution, physical causal inference, intention inference

## Abstract

Individuals with autism spectrum disorder (ASD) show impaired mentalizing skills, specifically in understanding intentions. They have difficulty understanding social situations with multiple cues due to their limited ability to perceive subtle social contextual cues. Studies that used comic strips and the strange stories as intention attribution tests found that individuals with ASD exhibit a reduced ability in attributing intentions compared to inferring causal consequences. This study aims to use static photographs of social scenes taken in everyday settings to investigate the ability of adolescents with ASD to infer intentions in social contexts, and to explore how intention inference relates to working memory and basic attention, including sustained attention, selective attention, and divided attention. The results show that the physical causal inference ability of adolescents with ASD is comparable to typically developing adolescents, whereas intention inference is notably weaker. Furthermore, working memory predicts physical causal inference and divided attention predicts intention inference in ASD.

## Introduction

Autism spectrum disorder (ASD) is characterized by core deficits in social cognition and interaction. A review comparing social and non-social cognitive deficits in adults with ASD indicated that impairments are strongest in the mentalizing domain (Velikonja et al., 2019). Mentalizing is a form of social cognition to comprehend both one’s own and others’ behaviors in terms of internal states, such as thoughts, feelings, desires, and goals (Luyten et al., 2020). Studies suggest that adults with ASD struggle significantly with their ability to infer the mental states of others (Happé, 1994; Baron-Cohen et al., 2001; Castelli et al., 2002; Mazza et al., 2022). Understanding others’ mental states (known as theory of mind) is a significant evolutionary achievement that deeply impacts our interactions with the world (Tomasello et al., 2005). Senju (2012) suggested two explanations for mentalization development failures in those with autism: one is that compensatory strategies may not be fully developed to allow for spontaneous and rapid mentalizing when needed, and the other is social cognition may not be automatically triggered by socially relevant cues.

Atypical social attention in ASD is widely discussed as a possible reason for difficulties in mentalizing. Social attention refers to the motivation, coordination of attention, and the focus on social cues within a contextual framework during interactions with others (Salley and Colombo, 2016). Individuals with ASD exhibit atypical attentional responses to social stimuli (Klin et al., 2002; Pelphrey et al., 2002; Corden et al., 2008; Norbury et al., 2009; Riby and Hancock, 2009a, 2009b; Nakano et al., 2010), including reduced attention to the eye and facial regions of others. In fact, when attending to faces, individuals with ASD tend to fixate more on the mouth area, and in situations presenting both social and non-social information concurrently, they may be more inclined to attend to body parts, objects, or stimuli in the background (Speer et al., 2007). Despite detecting similar information as neurotypical individuals, individuals with ASD demonstrate weaker sustained attention to social cues and a reduced ability to follow others’ gazes in social contexts (Fletcher-Watson et al., 2009; Freeth et al., 2010). Swettenham et al. (1998) proposes that the attentional patterns seen in individuals with ASD are more indicative of general attentional abnormalities rather than a deliberate avoidance of social stimuli.

Furthermore, adolescents with ASD have weaker abilities than typically developing peers in perceiving subtle social contextual cues (Hodgins et al., 2020), making it difficult for them to comprehend social situations that involve multiple social cues. Pierce et al. (1997) used videos depicting social interactions with 1 to 4 cues, including verbal, tonal, non-verbal with objects, and non-verbal without objects, to assess social perception in children with ASD, finding that they perform similarly to typically developing children on general attention tasks such as counting characters and identifying genders. However, their ability to interpret social perception questions, especially in scenarios that include multiple cues, is notably weaker than that of typically developing children. Relatedly, Vivanti et al. (2011) observed significant impairments in adolescents with ASD regarding their ability to attend to and interpret referential cues, such as interpreting a head turn to understand someone’s intentions. Contextual considerations alongside perceptual cues are crucial for judging interpersonal behaviors. Loveland et al. (2001) investigated how adolescents with ASD judge the appropriateness of social behaviors in videos containing multiple verbal and non-verbal cues. They found that adolescents with ASD are more likely to provide explanations of the protagonist’s behavior that are irrelevant or idiosyncratic to the context, whereas typically developing adolescents are more likely to interpret behaviors in accordance with social norms and principles.

Studies show that theory of mind ability and working memory are moderately correlated (Davis and Pratt, 1995; Gordon and Olson, 1998; Hughes, 1998; Keenan et al., 1998; Carlson et al., 2002; Meyer and Collier, 2020; Imanipour et al., 2021). According to Ericsson and Kintsch (1995), working memory is a subset of information drawn from long-term memory, distinguished by contextual markers that highlight its relevance to ongoing cognitive processing. Some researchers propose that children performing the theory of mind (false-belief). task must inhibit incorrect responses based on factual knowledge stored in working memory (Carlson and Moses, 2001; Leslie et al., 2004). Others suggest that children need to switch their attention between their own mental state and others’ mental states, both of which are managed in working memory (Frye et al., 1995; Andrews et al., 2003). Integrating both perspectives, with the ability to hold conflicting perspectives in mind, is essential for both learning and demonstrating knowledge related to theory of mind.

Social interactions rely on various interconnected processes such as attention, which is needed to interpret social cues and monitor others’ actions and intentions (Capozzi and Ristic, 2018; Dosi and Boni, 2023). These processes work together dynamically to facilitate effective social engagement. McGlamery et al. (2007) have concluded that attention is predictive of theory of mind scores for kindergarten boys. Basic attention is crucial for daily functioning and cognitive tasks in perception and learning, laying the groundwork for more advanced cognitive processes such as memory encoding and problem-solving. Capozzi and Ristic (2018) identified three core processes that collaborate with attentional systems to influence selective responses to the social environment: perception, interpretation, and evaluation. Perception helps in prioritizing social cues, interpretation connects attention to understanding the social significance of cues and others’ mental states, and evaluation assesses the value of social information sources. These processes collectively enable attention to effectively manage the vast amount of social information that one encounters daily.

Individuals with ASD show impaired mentalizing skills, specifically in understanding intentions, observed across both children and adults (Vivanti et al., 2011; Schneider et al., 2013; Schuwerk et al., 2016). They show reduced awareness of both their own and others’ intentions, which is linked to broader impairments in theory of mind (Williams and Happé, 2010). Bodner et al. (2015) used stories to elicit responses that described physical relationships and mental or emotional states. The study found that individuals with ASD may lack stored experiential knowledge needed for specific inferences, possibly due to linguistic limitations that hinder access to relevant experiences. Baron-Cohen et al. (1986) and Le Donne et al. (2023) used non-verbal comic strips to investigate intention attribution and physical causal inference in children and adults with ASD. The current study aims to investigate the ability of adolescents with ASD to infer intentions in natural social contexts, while mitigating the constraints posed by their linguistic limitations. The test materials used in the current study were static photographs of social scenes taken in everyday settings. The use of naturalistic test formats and stimuli in studying subtle mind-reading deficits related to autism can potentially enhance task sensitivity because these measures are designed to mimic the challenges of everyday social interactions, which may uncover difficulties in the real-time processing of mental states (Heavey et al., 2000). Some studies used photographs of social scenes featuring real people as testing instruments (Riby and Hancock, 2008; Rice et al., 2012; Harrop et al., 2019; Ioannou et al., 2020), but these studies investigated the visual social attention or orientation of individuals with autism. To the author’s knowledge, there have been no previous studies using photographs of natural social contexts to investigate characters’ intention and physical causal inference in individuals with ASD. Using real interpersonal interaction scenarios as testing instruments will be closer to real-life situations, and the results obtained will more accurately reflect the daily life intention reasoning ability of those with autism.

The current study also explores how intention inference relates to working memory and basic attention, including sustained attention, selective attention, and divided attention. Sustained attention is the ability to maintain focus over extended periods. Selective attention refers to the cognitive process of concentrating on particular stimuli while filtering out irrelevant or unattended input, allowing for focused processing of selected information. Divided attention explores the challenges of multitasking and the optimal allocation of cognitive resources between tasks, emphasizing the need to split or quickly shift focus due to the inability to process all information simultaneously (Parasuraman, 1998).

### Aims and research questions

The research questions of the current study explore the difference in intention reasoning abilities between adolescents with autism and typically developing adolescents, as well as the relationship between intention reasoning ability and working memory, sustained attention, selective attention, and divided attention.

## Materials and methods

### Participants

#### Pilot participants

The pilot participants consisted of 98 typically developing adolescents aged 11–18 years. Participants were recruited through school teachers who solicited willing participants from junior high and high schools. After distributing informed consent forms to students and parental consent forms, paper-and-pencil group tests were conducted at schools. Selection of schools took into account administrative district differences and gender ratios of participants.

#### Formal participants

The study included participants from an ASD group and a typically developing (TD) group. Recruitment for the ASD group was facilitated through autism foundations and their local branches nationwide, as well as by contacting schoolteachers to disseminate information. Inclusion criteria for the ASD group were as follows: (1) receiving a diagnosis of Asperger’s syndrome or mild ASD by a psychiatrist; (2) ages between 11 and 18 years and attending regular classes; (3) Full Scale IQ above 85 measured by Wechsler Intelligence Scale for Children, Fourth Edition; (4) no comorbidities such as attention deficit hyperactivity disorder, epilepsy, anxiety disorder, or depression. Recruitment for the TD group prioritized students in the same class or school as the ASD participants. Teachers helped exclude students with other disabilities and to control for age and gender ratios between the two groups at their schools. Finally, there were 32 participants in the ASD group with a mean age of 15.6 years and 33 participants in the TD group with a mean age of 15.5 years, with 4 females in each group.

The ASD group and the TD group were matched by the schools they attended to control for urban–rural disparities. The results of the socioeconomic status survey showed no significant differences between the ASD group and the TD group. Participants of both groups were born in Taiwan, and their parents are all of local nationality, with Mandarin being the primary language used.

### Procedure

The design of the test items involved referencing relevant research materials and collecting scenarios that align with the life experiences of typically developing adolescents aged 11–18 years. Real-life scenarios were captured using authentic photographs. Prior to the formal testing, the test items underwent content validity checks by two experts in the fields of psychology and special education. Subsequently, a pilot test was conducted using PowerPoint presentation software in a group setting, and based on the results, 4 items were removed. Each test item photo was displayed on a computer screen for 8 s.

The study was approved by the Institutional Review Board (IRB) of National Cheng Kung University in Taiwan. During the formal testing phase, both the TD group and the ASD group underwent individual testing. Participants were first given practice on 4 items, with the test administrator explaining the scenario depicted in each photo. The purpose of the practice was to ensure that participants understood the meanings of “accidently occurring” and “intentionally occurring.” If participants made incorrect judgments, the test administrator would reiterate the scenario and the correct answer. Practice continued until participants responded correctly. Formal testing involved the test administrator asking participants, after viewing each photo, whether the depicted scenario was “accidently occurring” or “intentionally occurring,” with the test administrator recording participants’ verbal responses for each item.

### Materials

#### Multi-dimension attention test

Multi-dimension attention test (Zhou et al., 1993) uses visual assessment materials to evaluate sustained attention, selective attention, and divided attention. Participants are required to circle corresponding items based on verbal instructions from the examiner under timed conditions. The test demonstrates a test–retest reliability coefficient ranging from 0.82 to 0.90 and an internal consistency coefficient ranging from 0.65 to 0.69.

#### Verbal working memory test

Verbal working memory test (Zeng, 1999) features common and high-frequency words from everyday life. Each item consists of 4 to 8 words. The examiner reads all words for each item aloud, provides an instruction, and asks participants to respond orally. For example, the examiner reads “desk, sausage, computer, dumpling, chicken leg and instructs, “Please verbally list the items that are edible in the original order.” The test presents a total of 18 auditory items. Test–retest reliability ranges from 0.65 to 0.82.

#### Intention inferences test

**Figure 1:**
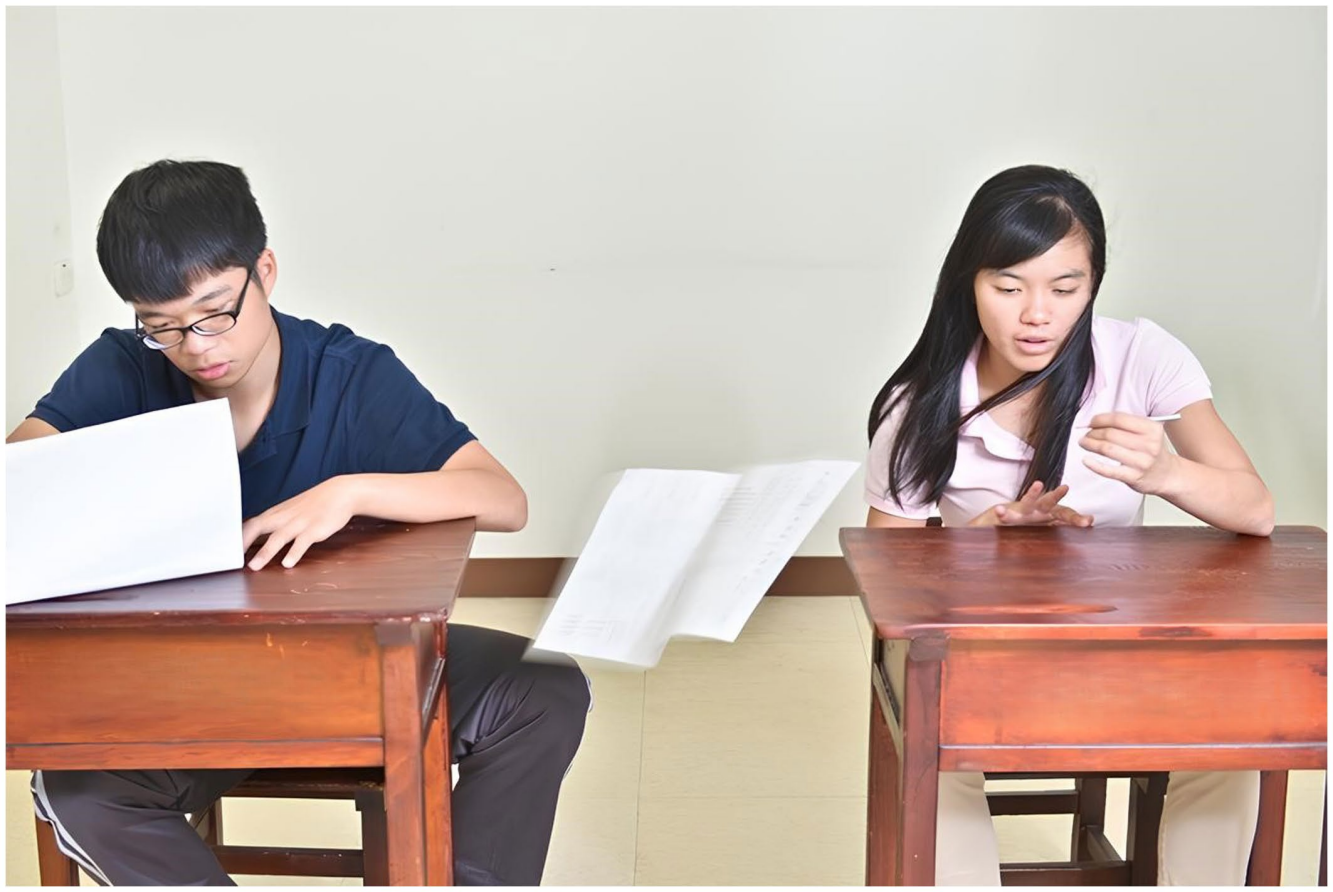
Sample of physical causal inference.

**Figure 2:**
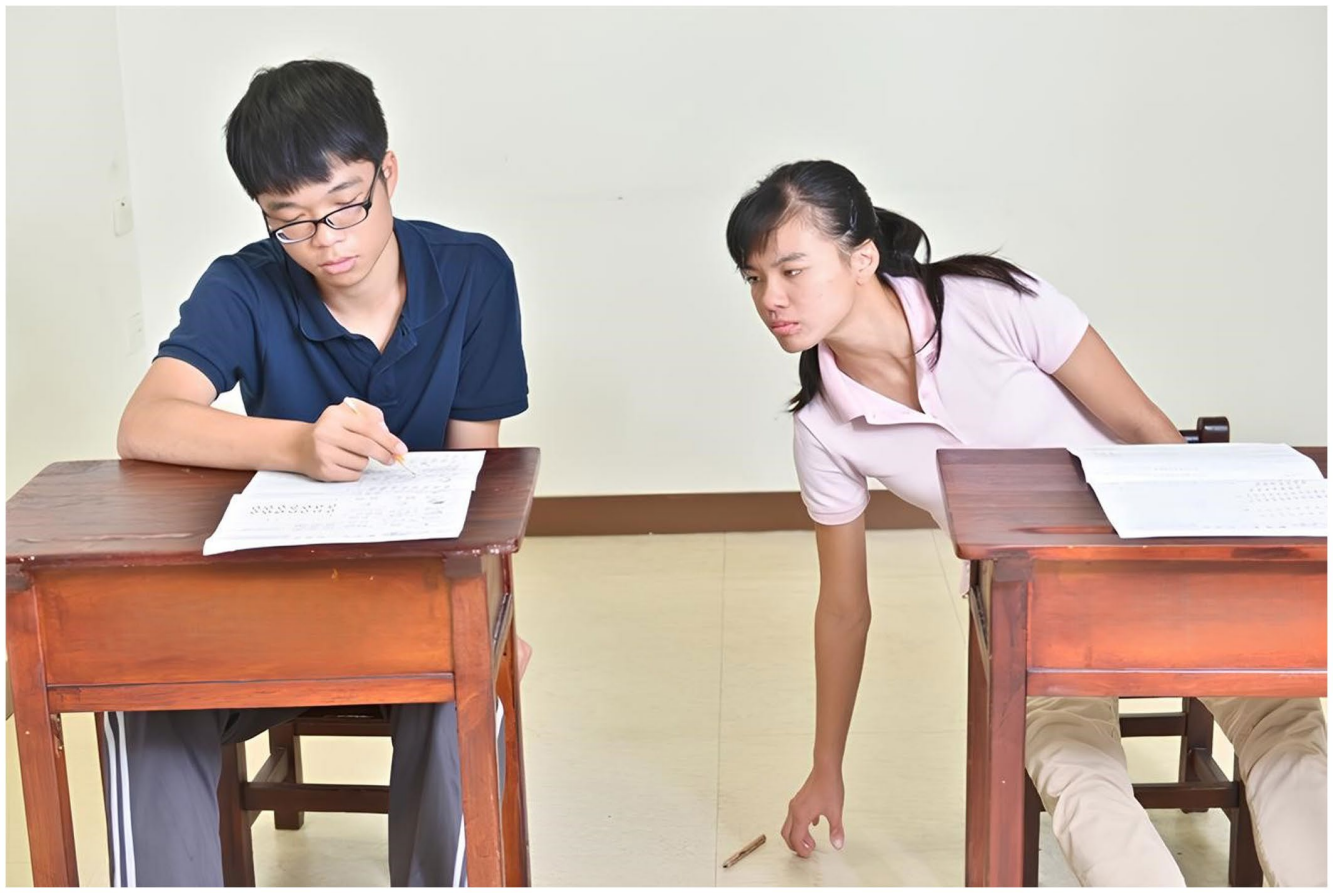
Sample of intention inference.

The content of the test items was derived from social scenes in daily life. Each item was presented with a color photograph, retaining the environmental background and devoid of dialog. Each photo depicted one male and one female, both without overt facial expressions and avoiding direct eye contact with the camera to prevent direct eye contact with the participants and potential influence (von dem Hagen et al., 2014). The number of objects appearing in all photos was limited to six or fewer. The items were categorized into two types: physical causal inference (Figure 1) and intention inference (Figure 2). The difference between the two lies in inferring whether an event or behavior occurred accidently or was deliberately caused by someone. There were 26 items in total, with 13 items for each category. Each set of physical causal inference and intention inference items had identical scenes, differing only in the interaction between the characters.

The results of the factor analysis of the pilot indicated that, after orthogonal rotation using the minimum oblique rotation method, the former could explain 30.02% of the variance, and the latter could explain 8.62%, totaling 38.64%. There was a correlation of 0.621 between the physical causal inference and intention inference factors, indicating a considerable relationship between the two factors. The Cronbach’s *α* coefficient among items was 0.912, indicating good reliability.

## Results

Table 1 presents descriptive statistics for physical causal inference and intention inference for ASD and TD groups. Table 2 shows the main effects of group differences reaching a significance level of 0.01, with a Partial Eta Squared of 0.199, indicating an effect size reaching the standard of Cohen’s moderate effect size. The overall mean scores for both physical causal inference and intention inference were significantly higher in the TD group compared to the ASD group. The difference in inference types reached a significance level of 0.001, with a Partial Eta Squared of 0.393, indicating an effect size reaching the standard of Cohen’s large effect size. Regardless of group, the mean score for physical causal inference was significantly higher than for intention inference. Furthermore, the interaction effect between inference types and groups reached a significance level of 0.001, with a Partial Eta Squared of 0.209, indicating an effect size reaching the standard of Cohen’s moderate effect size. As the interaction effect between inference types and groups reached significance, it is not appropriate to directly interpret the test results for the main effects of factors between and within groups. Instead, further analysis is required to examine the simple main effects of factors between and within groups.

**Table 1:** Descriptive statistics for physical causal inference and intention inference for ASD and TD groups.

Groups	*N*	Physical causal inference		Intention inference	
		Mean	SD	Mean	SD
ASD	32	12.47	0.63	10.37	1.83
TD	33	12.46	0.71	12.00	0.98

**Table 2:** Two-way mixed design ANOVA summary of groups and inference types.

Source	Type III sum of squares	df	Mean square	*F*	*p*	Partial Eta Squared
Between subjects						
Group (A)	18.46	1	18.46	13.42	0.001**	0.199
Ss w/in groups	74.31	63	1.18			
Within subjects						
Type of inference (B)	45.70	1	45.70	34.96	0.000***	0.393
A × B interaction	18.70	1	18.70	14.30	0.000***	0.209
B× Ss w/in groups	70.58	63	1.12			

***p < 0.001, **p < 0.01.

The results of the simple main effects ANOVA in Table 3 show that the difference between the two groups in physical causal inference did not reach a significance level, but the difference in intention inference did, with a Partial Eta Squared of 0.235, indicating an effect size reaching the standard of Cohen’s moderate effect size. The results of the simple main effects ANOVA pairwise comparisons revealed that in physical causal inference, the difference in mean scores between the TD and ASD groups did not reach a significance level. However, in intention inference, the mean score for the TD group was significantly higher than that for the ASD group, reaching a significance level. The results of the simple main effects MANOVA for each type of inference in both ASD and TD groups show that the difference in both types of inference reached a significance level in the ASD group, with a Partial Eta Squared of 0.484, indicating an effect size reaching the standard of Cohen’s large effect size. However, the difference in both types of inference did not reach a significance level of 0.05 in the TD group.

**Table 3:** Simple main effect ANOVA on physical causal inference and intention inference for ASD and TD groups.

Groups		SS	df	Mean Square	*F*	*p*	Partial Eta Squared	Compare means
Physical causal inference	Contrast	0.00	1	0.00	0.00	0.977	0.000	
	Error	23.93	63	0.38				
Intention inference	Contrast	37.16	1	37.16	16.50	0.000***	0.235	ASD < TD
	Error	120.97	63	1.92				

***p < 0.001.

**Figure 3:**
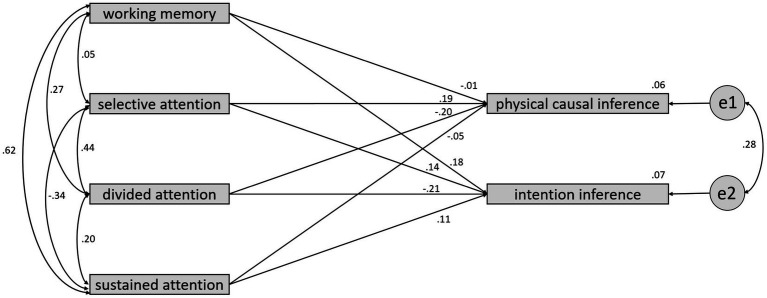
The regression coefficients between 4 variables and inference types for the TD group.

**Figure 4:**
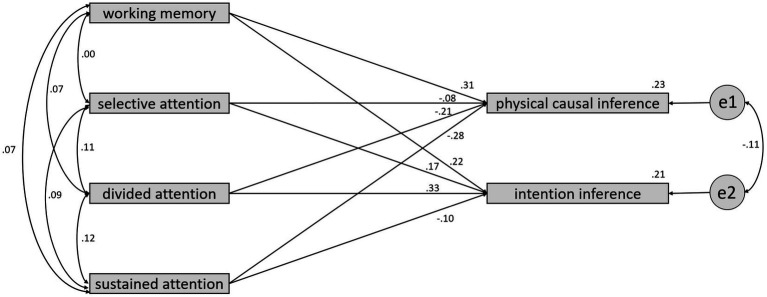
The regression coefficients between 4 variables and inference types for the ASD group.

In order to investigate whether there are differences in the predictive accuracy of physical causal inference and intention inference between the TD and ASD groups regarding four variables: working memory, selective attention, divided attention, and sustained attention, the current study conducted a multiple group analysis of structural equation modeling (SEM) to compare the differences in regression coefficients between the two groups. As shown in Figures 3, 4, the results of the significance tests for regression coefficients revealed that none of the coefficients reached the 0.05 significance level for the TD group. However, in the ASD group, the regression coefficient for working memory predicting physical causal inference reached a significance level of 0.05 in a one-tailed test, the regression coefficient for divided attention predicting intention inference reached a significance level of 0.05 in a two-tailed test, and the regression coefficient for sustained attention predicting physical causal inference reached a significance level of 0.05 in a one-tailed test.

Regarding the test for differences in regression coefficients between the two groups, divided attention had a standardized regression coefficient of −0.21 in the TD group and as high as 0.33 in the ASD group. The test for the difference in regression coefficients between the two groups yielded a critical ratio (CR) of 2.097, with *p* < 0.05. This result indicates that the largest difference in predictive models between the two groups is in the ASD group, where the regression coefficient for divided attention predicting intention inference is significantly higher than the TD group.

**Figure 5:**
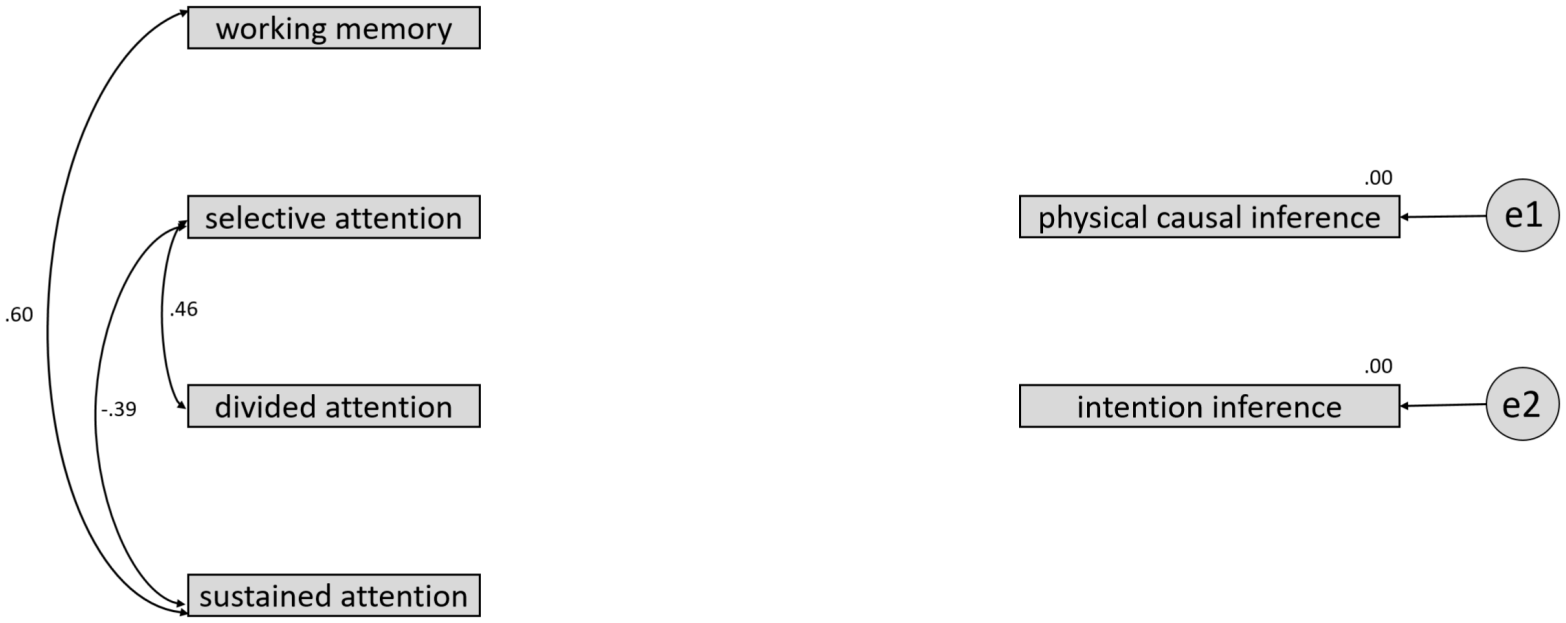
The most parsimonious model selected by the BCC for the TD group.

**Figure 6:**
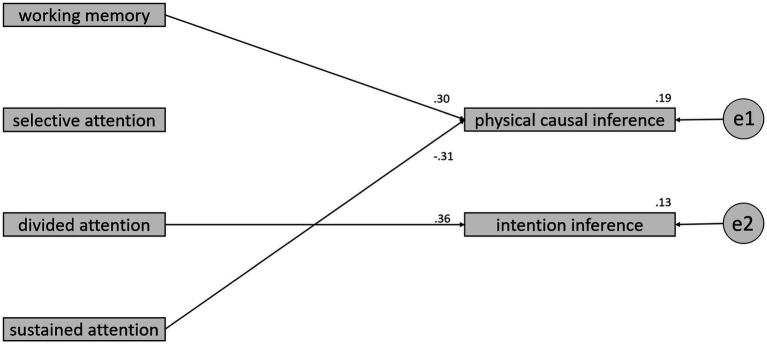
The most parsimonious model selected by the BCC for the ASD group.

The Specification Search method in the Amos software helps researchers choose a more concise and effective predictive model. In the current study, the Browne-Cudeck Criterion (BCC) was used to select the most parsimonious predictive model. The results, as shown in Figures 5, 6, indicate that for the TD group, the most parsimonious model retains only the correlations between predictor variables. The highest correlation observed is between working memory and sustained attention, reaching 0.60. The next highest correlation is between selective attention and divided attention, with a coefficient of 0.46. Notably, the correlation between selective attention and sustained attention is −0.39, suggesting that combining these two types of attention may not be suitable for the TD group.

The most parsimonious model for the ASD group is notably different from that of the TD group. According to the BCC, none of the correlations between the 4 variables were retained. However, the standardized coefficients for working memory and sustained attention predicting physical causal inference were 0.30 and −0.31, respectively, and both were retained. Additionally, the standardized coefficient for divided attention predicting intention inference was as high as 0.36, making it the largest of the 3 coefficients retained. The *R*-squared values for working memory and sustained attention predicting physical causal inference were 0.19, while for divided attention predicting intention inference, it was 0.13, both reaching the standard of Cohen’s moderate effect size.

## Discussion

The results show that the physical causal inference of adolescents with ASD in the current study is comparable to typically developing adolescents, but the intention inference is notably weaker. The results support the belief that high-ability individuals with ASD can understand causal-mechanical conditions, but have difficulties in mentalizing, or the ability to understand and think about other people’s thoughts and intentions (Baron-Cohen et al., 1986; David et al., 2008; Cole et al., 2018). Le Donne et al. (2023) used comic strips as an intention attribution test and found that the ASD group exhibited a reduced ability in attributing intentions compared to inferring causal consequences. In addition, studies using the strange stories tests have also shown consistent results in this direction (Happé, 1994; Jolliffe and Baron-Cohen, 1999; Kaland et al., 2005). As such, it appears that the use of different test content, including social scenes, comic strips, and the strange stories, have all produced the same result: compared to physical causal inference, individuals with ASD have remarkable difficulties in intention inference.

The social scenes used as test materials in the current study involve understanding the actions of the two characters in the photos. Understanding an action involves recognizing it on different levels: firstly, by identifying what was done and how; and secondly, by understanding why the action was performed and its effects (Kozak et al., 2006). Recognizing actions at the highest level suggests an awareness of one’s own mind as the cause of behavior (Levy et al., 2002). This ability, known as action identification, enables tracking and interpreting mental states, applicable to both oneself and others. Boria et al. (2009) discovered that children with ASD fail to recognize an agent’s actions on objects when those actions do not align with the standard use of the objects. Studies indicate that individuals with ASD process actions differently (Zalla et al., 2006; Vivanti et al., 2011; Kaiser and Pelphrey, 2012) and struggle with anticipating others’ actions and representing goal-directed behaviors (Zalla et al., 2006; Chambon et al., 2017).

Another finding of this study is that divided attention predicts intention inference in ASD. Understanding other people’s mental states involves a comprehensive analysis of their intentions, plans, personality, knowledge, emotions, beliefs, and desires. Additionally, it requires contextual understanding of the social situation in which events unfold (Killen et al., 2011). This holistic approach considers both individual psychological attributes and the broader social context as essential elements in understanding human behavior and interactions. The difficulties of people with autism are often reflected in terms of reduced attention to social cues in the environment (Leekam et al., 2000; Dawson et al., 2004; Shic et al., 2011). Our attentional system, typically involved in the pursuit of goal-directed behavior, serves the crucial function of selecting relevant stimuli and of ignoring irrelevant stimuli in different settings (Lavie et al., 2004). Frazier et al. (2017) suggest that in autism, failure to interpret people’s actions might originate from basic atypicalities in selecting, differentially attending to, and/or integrating relevant information. The result of the current study shows divided attention predicts intention inference in adolescents with ASD. It may be a discrimination problem in selecting the most salient stimuli, sustaining attention to the most salient stimuli, or of filtering out extraneous information during visual perceptual experiences (Frazier et al., 2017). Divided attention is an executive function that involves the central executive component of working memory, allowing individuals to manage multiple tasks or sources of information simultaneously (D’Esposito et al., 1995). Fisher and Happé (2005) conducted a training study of theory of mind, and found autistic children who received the executive functioning training program performed comparably to those who received the theory of mind training program in the post-test. Future research could explore whether divided attention training or executive functioning training can enhance the intention reasoning abilities of individuals with autism.

In the current study, an unexpected finding is that physical causal inference is negatively correlated with sustained attention in adolescents with ASD. Most studies which examined the sustained attention of people with ASD reported no deficits (Garretson et al., 1990; Buchsbaum et al., 1992; Casey et al., 1993; Minshew et al., 1999; Noterdaeme et al., 2001; May et al., 2015), or conversely, have noted heightened attention in those with ASD, particularly on topics or objects that interest them (Plaisted and Davis, 2009). A possible explanation for the result is that the attention of adolescents with ASD may focus on irrelevant cues (within the visual field/among the visual stimuli) or attend to relevant cues without abstracting the accurate interpretation. Klin et al. (2002) conducted one of the first eye-tracking studies which revealed that individuals with ASD show altered patterns of social visual engagement when observing natural social settings, and that they exhibit a decreased focus on eyes, but an increased attention toward mouths, bodies, and objects. Similarly, Chita-Tegmark (2016) reviewed eye-tracking studies and concluded that when attending to social stimuli, individuals with ASD spend less time looking at the core features of the face, eyes, and mouth, but spend more time looking at bodies and attending to non-social elements. In addition, attenuated sensitivity to peripheral social targets is found in autistic children (Hou et al., 2024). Observations from visual tracking and verbal reports of individuals with ASD further indicate a diminished ability to perceive social cues crucial for contextual understanding (Tassini et al., 2022).

Studies have demonstrated that individuals with ASD exhibit atypical patterns of selective attention in both social and non-social contexts. Dawson et al. (1998) observed deviations in selective attention related to social information processing in ASD, indicating challenges in attending to and processing social cues typical for neurotypical individuals. Additionally, Renner et al. (2006) and Keehn et al. (2010) found that individuals with ASD show atypical selective attention in non-social information processing. This suggests differences in how individuals with ASD allocate attention to and process non-social stimuli compared to typically developing individuals. However, the current study did not find any relation between selective attention and physical causal inference or intention inference in ASD. This requires further investigation in future studies.

The result of this study reveals that working memory predicts physical causal inference in ASD, supporting the correlation between working memory and theory of mind. Lecce and Bianco (2018) investigated the role of working memory in theory of mind changes during middle childhood and indicated that individual differences in working memory moderated improvement in children’s theory of mind. Accordingly, Lin et al. (2010) suggests that people with lower working memory capacity were less effective in applying their theory of mind to interpret behavior, and an attention-demanding task also reduced people’s ability to apply their theory of mind. To study theory of mind understanding, Gregory et al. (2002) and Zalla et al. (2009) adjusted their studies by accounting for the impact of detailed attention on working memory. This adjustment aimed to ensure that working memory limitations did not confound their findings on theory of mind. Those studies confirm working memory and attention play crucial roles when applying theory of mind for typically developing individuals.

## Limitations and future directions

The current study inevitably suffered from a range of limitations. This study used static photographs of social scenes to assess intention inference, which provided ecological validity. However, real-life social interactions are dynamic, and social cues appear rapidly, variables which cannot be accounted for in static photographs. It is suggested that future research consider using video clips of real social situations as testing materials to better approximate real-life contexts. This study did not test theory of mind or language abilities, so future research could analyze the relationship between theory of mind tests, language abilities, and intention reasoning. Since traits in female individuals with ASD may subtly differ from those in males (Hull et al., 2017), future research could include more females to investigate whether there are differences in intention reasoning abilities between genders in individuals with autism.

## Data Availability

The raw data supporting the conclusions of this article will be made available by the authors, without undue reservation.
